# Organoids: a systematic review of ethical issues

**DOI:** 10.1186/s13287-022-02950-9

**Published:** 2022-07-23

**Authors:** Dide de Jongh, Emma K. Massey, the VANGUARD consortium, Ekaterine Berishvili, Laura Mar Fonseca, Fanny Lebreton, Kevin Bellofatto, Juliette Bignard, Jochen Seissler, Leila Wolf-van Buerck, Mohsen Honarpisheh, Yichen Zhang, Yutian Lei, Monika Pehl, Antonia Follenzi, Christina Olgasi, Alessia Cucci, Chiara Borsotti, Simone Assanelli, Lorenzo Piemonti, Antonio Citro, Silvia Pellegrini, Cataldo Pignatelli, Francesco Campo, Olivier Thaunat, Antonia J. Cronin, Devi Mey, Chiara Parisotto, Giovanna Rossi, Patrick Kugelmeier, Petra Wolint, Markus Mühlemann, Karolina Pal-Kutas, Marco Cavallaro, Julia Götz, Jeanette Müller, Eline M. Bunnik

**Affiliations:** 1 Department of Nephrology and Transplantation, Erasmus MC, University Medical Centre Rotterdam, Rotterdam, The Netherlands; 2 Department of Medical Ethics, Philosophy and History of Medicine, Erasmus MC, University Medical Centre Rotterdam, Rotterdam, The Netherlands; 3 Department of Surgery, University of Geneva, Geneva, Switzerland; 4 Diabetes Centre – Campus Innenstadt, Medizinische Klinik und Poliklinik IV, Klinikum der Ludwig-Maximilians-Universität München, Munich, Germany; 5 Department of Health Sciences, University of Piemonte Orientale, Novara, Italy; 6 Diabetes Research Institute, IRCCS Ospedale San Raffaele, Milan, Italy; 7 Department Transplantation, Nephrology and Clinical Immunology, Lyon Claude Bernard University, Lyon, France; 8 King’s College, London, UK; 9 European Society for Organ Transplantation, Padua, Italy; 10 Kugelmeiers AG, Erlenbach, Switzerland; 11 Accelopment Switzerland Ltd, Zurich, Switzerland

**Keywords:** Organoids, Ethics, Research oversight, Informed consent, Personalized medicine, Transplantation, Brain organoids, Chimeras, Gastruloids, Stem cell research

## Abstract

Organoids are 3D structures grown from pluripotent stem cells derived from human tissue and serve as in vitro miniature models of human organs. Organoids are expected to revolutionize biomedical research and clinical care. However, organoids are not seen as morally neutral. For instance, tissue donors may perceive enduring personal connections with their organoids, setting higher bars for informed consent and patient participation. Also, several organoid sub-types, e.g., brain organoids and human–animal chimeric organoids, have raised controversy. This systematic review provides an overview of ethical discussions as conducted in the scientific literature on organoids. The review covers both research and clinical applications of organoid technology and discusses the topics informed consent, commercialization, personalized medicine, transplantation, brain organoids, chimeras, and gastruloids. It shows that further ethical research is needed especially on organoid transplantation, to help ensure the responsible development and clinical implementation of this technology in this field.

**Supplementary Information:**

The online version contains supplementary material available at 10.1186/s13287-022-02950-9.

## Background

An organoid is defined as a 3D structure, grown from clusters of organ-specific cells grown from pluripotent stem cells (PSCs), adult stem cells (stem cells taken from specific tissues), and somatic cells derived from human tissue. Organoids self-organize through cell sorting in a way that mimics complex structural and basic functional properties of a variety of specific organs or tissues [1–5]. The outcome is an in vitro miniature version of a human organ, that have similarities to the organ both in architecture and in physiology. Human organoids have been successfully generated for a wide range of organs, including the gut [6, 7], lung [8], thyroid [9], gastric [10], heart [11], kidney [12, 13], liver [14], brain [15–19], and retina [20]. These mini-organs can be stored in biobanks and used for fundamental research, such as disease modeling and developmental biology, but also for translational research, such as drug screening. Moreover, the potential clinical applications of organoids are manifold, including personalized and regenerative medicine. As an example, patient-derived gut organoids developed from cystic fibrosis (CF) patients have served as personalized drug-testing tools and created possibilities to develop personalized treatment (or precision medicine) for CF patients [3, 7, 21–23]. Another example of a clinical application is the use of organoids in regenerative medicine, as a source of potential functional tissues for transplantation in human patients [23, 24].

While organoids thus show promise for both research and clinical purposes, there are many technical limitations to overcome [21–23, 25–29]. For instance, organoids lack the directional cues required for mature tissue architecture and are not (yet) capable of growing the size of a real organ. They require, but cannot self-generate, nutrition and vascularization, and lack essential organ-specific cells (especially immunological cells, which are part of the immune system and help the body fight infections and other diseases). Furthermore, their production is heterogenic and inconsistent, which induces organoid–organoid variability and problems with upscaling. If these hurdles can be overcome, organoids may deeply impact biomedical research and clinical care. In addition, organoids can be seen as scientifically more accurate and ethically more acceptable alternatives to animal and embryo models, which have traditionally been used in research [30]. Organoids might be better models than animals, for instance, for the purposes of drug screening, because they are grown from human, even patient-specific, cells and tissues. Moreover, by using organoids instead of animal models, fewer laboratory animals will be needed [30], which is in line with the ethical principle of ‘reduction’ of animal experiments. Likewise, for some research purposes, organoid technology can replace the—ethically contentious—use of embryos. Nevertheless, organoids should not be seen as an ethically neutral alternative. Organoids are grown from cells and tissues obtained from human individuals, and this connection between organoids and humans gives rise to ethical concerns at the level of the organoids themselves, of the individual patient or donor, and of society at large.

Since the early 2010s, rapid improvements in organoid research and clinical advancements have been made, which have been widely discussed in the academic and scientific literature. However, a systematic review of the ethical issues surrounding organoid technology have not yet been published. A systematic review can be useful as a reference work for clinicians, researchers, policymakers, and ethicists who are interested in ethical issues surrounding organoid technology. The purpose of the present systematic review is to provide an overview of ethical issues related to the use of organoids in research and clinical settings. It collates and categorizes all ethical themes related to organoids as discussed in the literature and shows how much attention has been paid to each theme. Thus, this systematic review identifies gaps in the literature and sets an agenda for future ethical work on organoid technology.

## Methods

In contrast to systematic reviews in empirical disciplines, there are currently no guidelines or manuals on how to conduct systematic literature reviews of bioethical topics, only some published suggestions [31–35]. Therefore, we followed the PRISMA statement, as far as applicable (see Additional file 1) [36, 37]. The review protocol has not been published or registered.

### Search and selection strategy

**Fig. 1:**
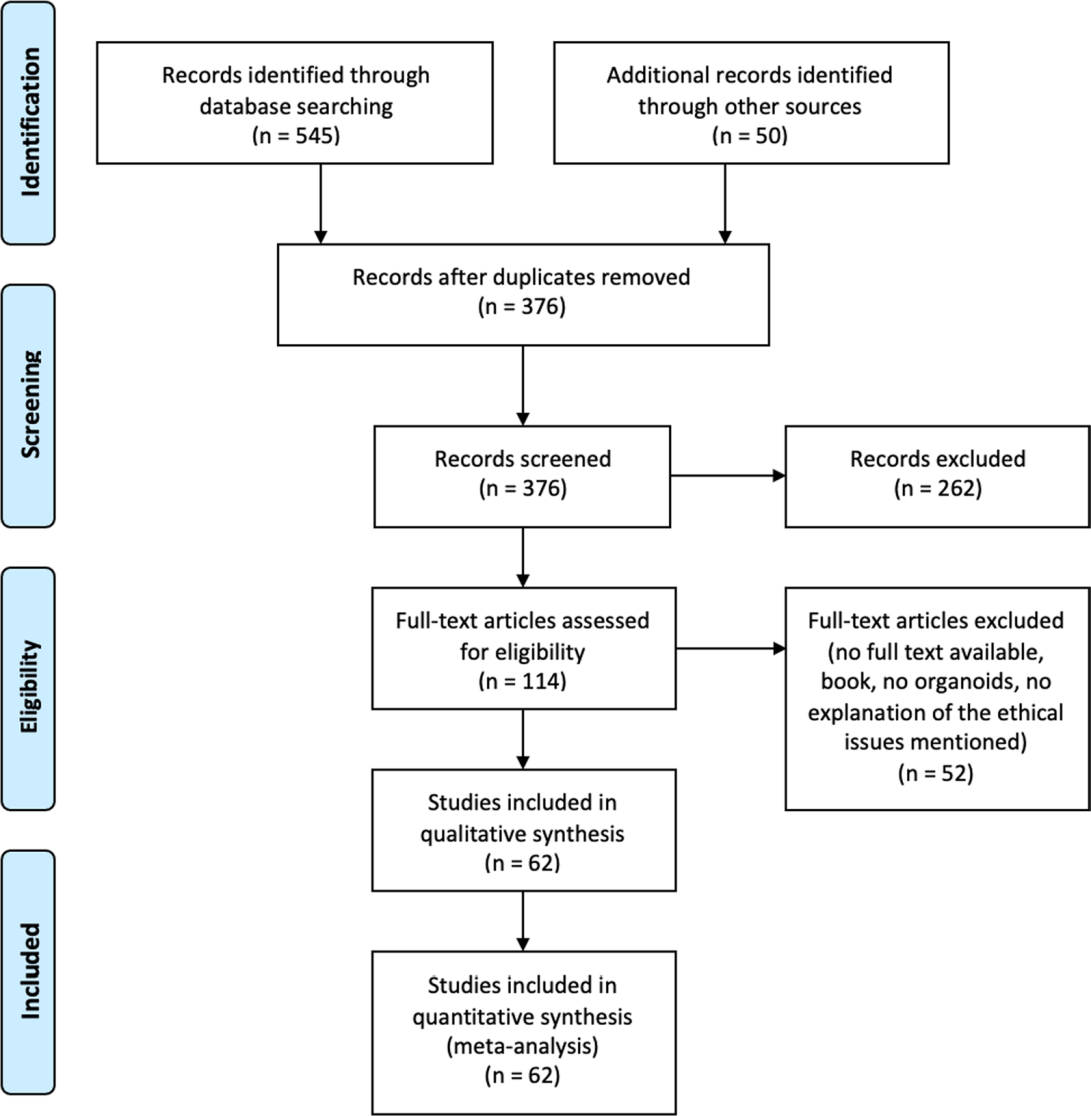
A PRISMA flow diagram of the included literature

We (DJ, EB, and EM) developed the search strategy with support from a university librarian. We conducted the literature search in June 2021, using the following seven bioethics and biomedical databases: PubMed, EMBASE, Medline, Web of Science Core Collection, Cochrane Central Register of Controlled Trials, and PsycINFO. An additional systematic search of the grey literature was conducted in Google Scholar. Search strings have been constructed by keywords and their truncation, and relevant database-specific subjects headings [MeSH terms] targeting ORGANOIDS and ETHICS have been used (see Additional file 2). Because of language barriers, only articles in English, German, or Dutch were considered for full-text analysis. We screened all titles and abstracts on our subject until June 2021 with no restriction for date of publication. At this stage, based on title and abstract, the articles that fulfilled the inclusion/exclusion criteria were selected. The selection was carried out blinded by two researchers (DJ and EB). Discrepancies about meeting the inclusion criteria were resolved through retrieval of the full text and via consensus-seeking discussions between the researchers (DJ, EB, and EM). After title and abstract selection, full texts were screened. Finally, the reference list of the full-text selected articles was checked for possible missed scientific articles or other documents and included when inclusion/exclusion criteria were fulfilled (DJ) (see Fig. 1).

### Inclusion and exclusion criteria

All articles that mentioned and described ethical issues, questions, or challenges related to organoids were included. Accordingly, letters to the editor, editorials/commentaries, case reports, and highlighted news stories within a scientific journal were included as non-research manuscripts. However, biomedical articles that only focused on the technical development of organoids and did not mention any ethical issues were excluded from our sample. Likewise, abstracts for conferences and societies were excluded. All articles that met our inclusion criteria are listed in Table 1.

**Table 1:** Themes addressed in the included articles

Author	Journal	Country	Year	Informed consent	Commercialization	Personalized drug testing	Transplantation	Brain organoids	Chimeras	Gastruloids
Aach et al.	eLif2	UK	2017					x	x	xx
Ankeny et al.	The American journal of bioethics	Australia	2020					xx		
Bayne et al.	Trends in neuroscience	Australia/UK/Italy/Canada	2020					xx		
Birch et al.	The American journal of bioethics	UK	2020					xx		
Bitar et al.	Frontiers in molecular neuroscience	Australia	2020					x	x	
Blue et al.	Neurosurgery	USA	2020	x	x			x	x	
Boers et al.	EMBO rep	The Netherlands	2016	xx	xx	xx	xx			
Boers et al.	Nature Cell Biology	The Netherlands	2018	xx	xx			x	x	
Boers et al.	Journal of cystic fibrosis	The Netherlands	2018	xx	xx	xx				
Boers et al.	Journal of medical ethics	The Netherlands	2019	xx	xx	xx	xx	x	x	x
Brazzanella	BioLaw journal	Italy	2021					x		
Bredenoord et al.	Science	The Netherlands	2017	xx	xx	xx	xx	x		x
Buchanan	Nature Physics	USA	2018					x	x	
Chen et al.	Developmental dynamics	USA	2019	x		x	x	x	x	
Chen et al.	Cell stem cell	USA	2019	x				x	xx	
Chhibber et al.	Drug discovery today	USA	2020	x	x			x	x	
Choudhury et al.	Trends in molecular medicine	Singapore	2020	x	x				x	
Cleber et al.	Trends mol med,	USA	2018					x		
Denker	Cells	Germany	2021							xx
Farahany	Nature	USA	2018	x	x			xx	xx	
Felsen et al.	The journal of law, medicine and ethics	USA	2019					xx		
Greely	The American Journal of bioethics	USA	2020	x				xx	xx	
Haselager et al.	Regenerative medicine	The Netherlands	2020	xx	x	xx		xx		
Hostiuc et al.	Regenerative therapy	Romania	2019					xx	xx	x
Hyun et al.	Brain research	USA	2020	xx	x	xx	x	xx	xx	
Hyun et al.	Cell stem all	USA	2017	x	x	x		x		
Kamm et al.	APL Bioengineering	USA	2018					x		x
Koplin et al.	Journal of medical ethics	Australia	2020	x				xx	xx	
Koplin et al.	Journal of law, medicine and ethics	Australia	2019	x				xx	x	
Koplin et al.	Monash Bioethics Review	Australia	2020					xx	xx	
Lavazza	Journal medical ethics	Italy	2019	xx	xx			x	x	
Lavazza	Brain research	Italy	2020					xx		xx
Lavazza et al.	Journal of law and the Bioscience	Italy	2020					xx	x	
Lavazza et al.	Neuroethics	Italy	2018		x			xx	x	
Lavazza et al.	Medical journal of medical ethics	Italy	2018	x	x			xx		
Lavazza	Monash Bioethics review	Italy	2020					xx	xx	
Lensink et al.	Development	The Netherlands	2020	xx	xx	x		x	x	x
Lensink et al.	Personalized medicine	The Netherlands	2020	xx	xx	xx				
Lensink et al.	Journal of cystic fibrosis	The Netherlands	2020	xx	xx	xx				
Li et al.	Biopreservation and biobanking,	China	2020	x	x			x		
Lunshof	The American journal of bioethics	The Netherlands	2020					xx		
Lyon et al.	Brain	UK	2019					x		
Munsie et al.	Development	Australia	2017	xx	x			x	xx	xx
Munsie et al.	Genetics and development	Australia	2018	x	x			x	xx	x
Ooi et al.	The neuroscience	Australia	2020	x				x		
Pera et al.	Nature methods	Australia	2015							xx
Pereira Daoud et al.	Human reproductive update	The Netherlands	2020							xx
Reardon	Nature		2018					x		
Reardon	Nature	USA	2020					xx		
Rinaldi	Future medical chemistry	Italy	2018	x		x	x	x		
Rivron and Pera et al.	Nature	The Netherlands/USA	2018							x
Sample	Biofabrication	Canada/USA	2019		x				x	
Sawai et al.	Stem cell reports	Japan	2019					xx	xx	
Sawai et al.	AJOB neuroscience	Japan	2021	x				xx	xx	
Schneeman et al.	Science translational medicine	The Netherlands	2020				xx			
Sharma and Zuk et al.	The American journal of bioethics	USA	2020					xx		
Shepherd et al.	Journal of medical ethics	Canada	2018					xx		
Sugarman and Bredenoord	Science and society EMBO reports	USA/The Netherlands	2020	x	x		x		x	x
Sugarman and Bredenoord	Cell stem cell	USA/The Netherlands	2019	xx	xx	xx	xx	xx	xx	xx
Wei et al.	Reproductive science	China	2021	x					x	x
Xinaris et al.	Current opinion organ transplant	Italy	2019					x		
Zuradzki	The American journal of bioethics	Poland	2020					x		
**Total**				**31**	**23**	**13**	**9**	**49**	**30**	**16**

x: Mentions the theme as an ethical issue

xx: Ethical issue is elaborately discussed in the paper

### Analyses and syntheses

The method of qualitative content analysis was employed [38]. Qualitative content analysis is an inductive approach to categorize ethical issues and to develop sub-themes of issues within a coding frame. One researcher (DJ) conducted the analyses. Firstly, codes were assigned to all the ethical issues mentioned in each publication. Secondly, themes and sub-themes were created out of these codes by DJ. Thirdly, EM and EB checked whether the themes and sub-themes developed by DJ were correct and logically created out of the codes and whether overarching themes were missing. Fourthly, the words which described the themes and sub-themes were discussed (by DJ, EM, and EB) until agreement was reached. Finally, a coding frame was built out of the identified themes and sub-themes. As an example, we distinguished ‘ethical issues when organoids are used as research applications’ as a theme and ‘challenges of informed consent’ as a sub-theme of ‘ethical issues when organoids are used as research applications.’ The coding frame was mainly used to systematically keep track of the identified themes and sub-themes per included article and to identify and summarize all the ethical issues mentioned per theme.

### Quality appraisal

No quality appraisal procedure has been assessed, because of the lack of suitable or applicable criteria to appraise the quality of the literature included. This is a well-documented limitation of systematic reviews of ethical literature [39–41]. Instead, assessments were made of the *extent* to which ethical themes mentioned in the included papers were *discussed* in those papers (DJ). For each included paper, in Table 1 it was assessed whether the theme is either merely flagged or briefly mentioned as an ethical issue in the paper, or discussed more elaborately, within the paper.

## Results

A PRISMA flow diagram has been made of the selection procedure; see Fig. 1. The search produced 376 hits, of which 114 were deemed eligible based on title or abstract and 62 were included after reference check and full-text screening. The publication year ranged from October 2015 to June 2021.

### Themes

First, we distinguished two different sets of applications of organoid technology: the use of organoids in research settings and the use of organoids in clinical care, each raising their own sets of ethical issues. Below, we will first discuss research applications of organoids and present two sub-themes of ethical issues, i.e., the challenges of informed consent and concerns regarding the commercialization of organoids. Then, we discuss clinical applications and present the sub-themes personalized medicine and organoid transplantation. Lastly, we discuss ethical issues that are associated specifically with specific sub-types of organoids: brain organoids, chimeras, and gastruloids. These organoids raise specific ethical questions not seen in other areas of organoid research, especially questions that are related to consciousness and the moral status of these entities. For each included paper, Table 1 shows which themes are mentioned in the paper, and whether these themes are either only mentioned in passing or discussed more elaborately within the paper.

### What are the ethical issues when organoids are used in research?

The ethical literature on the use of organoids in research mentioned two prominent issues: developing an informed consent model for donors participating in organoid research and the potential to commercialize organoids.

#### Challenges of informed consent

A frequently discussed ethical issue is informed consent—whether, and what kind of, consent is required. In total, thirty-one articles emphasized the importance of an appropriate informed consent model for tissue donors participating in organoid research. Eighteen articles either did not elaborately discuss this issue [21, 22, 42–56] or focused only on consent for the use of organoids for one specific disease, such as dementia [28]. Thirteen articles discussed—albeit concisely—ethical issues related to informed consent [30, 55, 57–67]. Our search strategy identified four empirical papers which investigated the perspectives of tissue donors on informed consent for research in which organoids are generated [55, 61, 66, 67].

### The de-identification approach in organoid research

A traditional rule of thumb for the secondary use of human tissue for research purposes is ‘consent or anonymize’ [30]: Researchers should either obtain the consent of a donor or de-identify the sample. When tissue is completely de-identified, it is felt that the interests and privacy of donors are adequately protected [60]. However, multiple articles in our sample argued that today, complete anonymity of human tissue is neither possible, nor desirable, due to the following three reasons [30, 57–62, 68]. Firstly, whether absolute anonymity of human tissue is possible in organoid research is considered questionable given the current developments in big data research and genomics. In the case of organoids, data (re)identification is especially relevant, because organoids could be made out of human tissues from donors with rare mutations and/or diseases, like cystic fibrosis (CF) [57, 59, 62, 68]. Therefore, the anonymity of these donors cannot be guaranteed. Secondly, complete de-identification is not desirable, because de-identification makes organoids scientifically and clinically less useful. For instance, when the coupling of organoids with personal and biological data is missing, organoids are unsuitable for precision medicine [30, 59, 62, 67, 68], as diagnoses, drug possibilities, or other relevant research results cannot be returned to donors. Not only will organoid research be less useful for patients, it will also not be possible, for instance, to validate prediction models based on data generated from organoids. Moreover, when donors cannot be followed on the long-term, valuable data will be missed. As a consequence, the utility of the organoid as a model decreases when data are de-identified. Lastly, an ethical reason to not anonymize donor tissue in organoid research is that the donor will be unable to control and/or manage the subsequent use of their samples, as they lose the opportunity to withdraw, which is particularly important when controversial organoids are made out of their tissues [55, 57–62, 66–68]. Two interview studies of CF patients in the Netherlands showed that tissue donors have a wish to control the research use of organoids derived from their tissues [61, 66]. For instance, patients in these studies desired to be informed about the results of research conducted with their tissue or about arrangements made between biobanks and commercial partners regarding profits and drug pricing [61, 66]

### Challenges of informed consent in organoid research

If anonymization is less desirable or impossible to achieve, an appropriate informed consent model is necessary. The literature offered five reasons why an informed consent model is ethically and practically challenging to accomplish in organoid research. Firstly, research and technological opportunities are changing fast and the potential clinical applications for organoids are unknown, which could make it difficult to foresee and describe—as part of the informed consent process—the possible future uses and storage of donor samples [30, 55, 59, 61–63, 65–67, 69]. For instance, studies showed that donors are cautious regarding potential future technological applications and possibilities, such as cloning, transplantation, and human enhancement [61, 66]. Some respondents felt that using organoids for the purposes of human enhancement should not be allowed [66]. Secondly, many parties are involved in the processes of generating organoids for research, which makes it difficult to protect and balance the values, interests, and long-term engagement of all parties (researchers, companies, patients, and donors) involved in an informed consent procedure [30, 55, 59, 60, 62, 64, 66–69]. Particularly, the combination of organoid research applications and the clinical use of organoids makes informed consent procedures more difficult [62, 67]. For instance, organoid biobanks have as a main goal to generate scientific knowledge, and companies may be involved to commercialize organoids generated from donor tissue, whereas patients are mainly interested in the utility of organoids to improve their health status or that of other patients [61, 66, 67]. Thirdly, donors may relate differently (i.e., more strongly) toward complex organoid models generated from their tissue than to cell lines when it comes to body integrity and identity, which could call for a more demanding informed consent procedure [30, 55, 59, 60, 62, 66–68]. For instance, Boers et al. [30] showed that organoids derived from donor tissue are experienced by donors as an entity with close and distant ties to their personal identities, often at the same time, which indicates that donors have ambiguous ideas about the closeness between human bodies and organoids. Fourthly, tissue taken from donors can be used to generate organoid sub-types that are seen as ethically sensitive, such as brain organoid [55, 66]. For instance, respondents from the study by Haselager et al. [55], who interviewed 28 patients and laymen regarding the development, use, and storage of brain organoids, indicated that the respondents were concerned that brain organoids would reveal personal aspects of the donors, such as their emotions; such misunderstandings might render informed consent more complex. Finally, a link between the donor and the sample is of clinical relevance in organoid research, but complicates privacy protection [55, 59, 66–68]. The fact that the risk of traceability and potential consequences are often unknown suggests that these aspects need to be incorporated more adequately in informed consent processes.

### Informed consent models for organoid research

Several informed consent models have been proposed and discussed for organoid research: specific, tiered, broad, blanket, opt-in, opt-out, governance, and dynamic consent [30, 59–62, 67, 68]. Although an in-depth review of all the different proposed consent types is beyond the scope of this review, Table 2 presents a short summary of the consent models described in the literature and their advantages and disadvantages.

**Table 2:** Advantages and disadvantages of consent models proposed for organoid research explained in the literature

Type of consent	Summary of the model	Advantages	Disadvantages
Specific consent and re-consent [59, 61, 62, 68]	Donors consent to the use of their tissue for a specific research project and are re-contacted to provide consent for each new potential future study that will be conducted with their tissue		Donors are re-contacted for each scientific (re)use of their sample. Enable to engage the preferences of the donors
Tiered consent [59, 60, 66, 67]	Donors are presented with a list of specific research projects and given the opportunity to provide or withhold consent for specific uses of their tissue	Able to engage the preferences of the donors	Donors are re-contacted for each scientific (re)use of their sample
Broad consent [30, 59, 61, 62, 67, 68]	Donors consent to a broad range of future research purposes, the specific details of which are unknown at the time of consent	Donors are not re-contacted for each scientific (re)use of their sample	Enable to engage the preferences of the donors
Blanket consent [59]	Donors consent to the use of their samples for future research without restrictions	Donors are not re-contacted for each scientific (re)use of their sample	Enable to engage the preferences of the donors
Opt-in [30, 59, 60]	Donors consent explicitly before their samples can be used for scientific research		Donors are re-contacted for each scientific (re)use of their sample. Enable to engage the preferences of the donors
Opt-out [30, 59, 60]	Donor consent is implied, unless the donor explicit refuses to use their biomaterials	Donors are not re-contacted for each scientific (re)use of their sample	Enable to engage the preferences of the donors
Governance consent [30, 59, 62, 68]	Donors consent to governance obligations in the organoid infrastructure to which they contribute. Donors do not exactly know in which studies their tissue will be used, but they do know how researchers will protect their privacy and interests	Able to engage the preferences of the donors. Donors are not re-contacted for each scientific (re)use of their sample. Ongoing communicative (governance) process	
Dynamic consent [59, 60, 62, 67]	Facilitates a two-way communication between donors and researchers through the use of digital interfaces	Able to engage the preferences of the donors. On-going communicative (governance) process	Donors are re-contacted for each scientific (re)use of their sample

Blanket consent, opt-out, broad consent, and specific consent are different approaches to the scope of the future uses of (the type of research that will be conducted using) organoids. In specific consent, donors consent to a specific research project, which is in stark contrast to blanked consent and opt-out, in which donors consent to an open-ended future use of their tissue. In broad consent, participants are asked to consent to a (broad) category of future research uses (e.g., organoid research in a particular disease area); it is less explicit in providing specific details to patients than specific consent, but narrower than blanket consent. Moreover, while all consent procedures offer participants the opportunity to refuse further use of their biomaterial for scientific research, they differ in the level of control and the extent of information provided to participants. In specific, tiered, opt-in, and dynamic models, participants are commonly (re-contacted and) asked for explicit consent for each scientific (re)use of their samples specifically. However, in broad, blanket and governance consent and opt-out models, samples may be (re)used for a range of research projects without (re)contacting the donor with information about those projects. While in four papers, re-contacting the donor for each new research application is seen as ethically appropriate, it is not seen as feasible [61–64]. Furthermore, three of these papers mentioned that re-contacting could lead to an overload of information for donors, which could undermine efforts to obtain informed consent [61–63]. Some participants in the study by Lensnink et al. [67] emphasized that re-contacting implies an investment of time, costs, and resources, which could eventually lead to hampering research. According to the empirical study by Boers et al. [61], most tissue donors in organoid research consider broad consent as an insufficient model for organoid research, because of its inability to incorporate their concerns, values, and hopes about organoid research into the consent model [61]. The dynamic, governance, and tiered consent models focus on stimulating the control and engagement of donors and emphasize a more active model of involvement, rather than passive donation. This is achieved by facilitating ongoing communication with the donors and providing them with some form of representative power that could potentially improve accountability, trust, and willingness among donors to participate to research and could bring them in a position to negotiate collective interests vis-à-vis their organoids with other stakeholders [59–62, 66–68]. Tiered consent for organoid research is often brought up by patients [66] and professionals [67]. The consent of governance and dynamic models see consent not as a one-off event but as an on-going communicative (governance) process. In the governance model, for example, donors do not exactly know in which studies their tissue will be used, but they do know how researchers will protect their privacy and interests [55, 59, 68]. The study by Haselager et al. [55] emphasized that patients trust that good governance will ensure their privacy and safety.

#### Concerns regarding commercialization of organoids

Asking for informed consent is not the only condition for doing ethically responsible organoid research. Ethical conditions or guidance with regard to commercialization is also important, which is mentioned in twenty-three articles [64]. Twelve of the articles mentioned the commercialization of organoids as an ethical challenge [21, 42–45, 48, 49, 51, 53, 56–58, 69], but did not discuss it further. Ten articles concisely discussed the ethical challenges regarding organoid commercialization [30, 55, 59–64, 66, 68]. When technologies to transform donor cells into organoids are considered scientifically and clinically promising as well as sufficiently novel, they could be patentable [60, 68]. Thus, parties other than donors could obtain property rights over organoids or organoid technologies. At the moment, the commercial value of organoids is rapidly increasing for both public and private stakeholders, including researchers groups and businesses, such as pharmaceutical companies [25, 53, 59–61, 68]. Profit generation of organoids by commercial parties could lead to advancements in science, which is considered hopeful by patients and professionals [55, 66]. At the same time, patients are concerned about close commercial involvement in organoid biobanking [55, 66]. These concerns have been linked to the negative reputation of pharmaceutical companies regarding drug pricing, sharing data on research participants and excessive profit generation [55, 66]. Profit-making out of organoids may be seen as problematic by patients, donors, and other stakeholder groups, because of three reasons. Firstly, the notion of profit-making creates tensions between the altruistic motivations of donors and the interests of commercial parties. While donors donate their tissue altruistically and without receiving any benefits, commercial parties receive money out of ‘their’ genetically linked organoids by establishing a form of property rights by transforming organoids into marketable products to sell to third parties. Because of this tension, ten scientific articles emphasize the importance of fair distribution of benefits between commercial parties, researchers, donor, and other stakeholders [55, 57, 59–64, 66–68]. Seven papers in our sample made it explicit that such benefits to donors should be non-monetary rather than monetary and may include access to generated knowledge, clinical benefits, or early access to novel therapies that are developed through the usage of ‘their’ organoids [57, 59, 60, 62, 66–68]. Additionally, it is suggested that commercial parties should reinvest the profits back into research projects or use them to pay for post-trial access to increase donor trust and fairness.

Secondly, generating profits using tissues derived from patients is considered ethically contentious and unfair, more so than from healthy donors, because patients are in a dependent position [62, 66, 67]. The patent owner, which could be a commercial entity, has the right to grant or deny access to organoids and to set prices for their use in personalized medicine [68]. Indeed, patients are dependent on the development of drugs by commercial parties. For instance, patients in the study by Lensink et al. [66] emphasized that their personal stake in organoid biobanking consisted of a personal dependency on innovative research to improve their health. In relation to this, some articles highlighted that when organoids are used for drug testing, proactive measures should be taken to ensure that patients will have (early) access to innovative therapies with fair prices and the return of individually relevant research results [57, 59, 62, 66]. Lastly, the commercialization of organoids could raise ethical concerns among donors because of the experienced close relational value of donors toward their genetically linked organoid [55, 61, 62, 66–68]. The explanation of the close relational value of donors toward ‘their’ organoids seems partly due to the 3D structure of an organoid, which distinguishes organoids from other immortalized cells. For instance, (potential) donors have recognized organoids as living human materials or living fragments of themselves and felt a connection with the organoids [55, 61]. Three empirical studies emphasized the importance of specifying ownership, because of the commercial value of organoids and their genetic and functional links with the donor [55, 66, 67].

### What are the ethical issues when organoids are used in the clinic?

The ethical literature on clinical applications of organoid technology is mainly focused on two applications: personalized medicine (in vitro) and transplanting organoids in humans (in vivo).

#### Organoids in personalized medicine

Patient-derived organoids can be used for drug testing [22], allowing for personalized medicine. As an in vitro model of an individual patient, the organoid can be used to predict the effectiveness of a drug in that patient. For this reason, the organoid is seen as a new type of evidence for the clinician, which can be used for treatment selection [63]. When the effectiveness of drugs can be predicted using organoids, patients no longer need to be exposed to drugs that are unlikely to work and may have side effects [63]. A current example is the treatment of cystic fibrosis (CF) with the new, expensive drug ivacaftor, which is prescribed and reimbursed conditional upon on a positive response in a patient-derived gut organoid [57]. Besides the benefits of patient-derived organoids for individual patients, seven different ethical concerns are highlighted in thirteen scientific articles [22, 30, 43, 52, 55, 57, 60–63, 66–68]. Firstly, when organoids are used as a tool to select or develop personalized treatment, it is critical to maintain linkages between the patient and their organoids to return valuable results, while safeguarding their privacy [30, 60, 62, 63, 66–68]. Secondly, it takes a long time to generate an organoid and the cost of developing personalized medicine with the use of patients-derived organoids is high, which is a major practical impairment to the use of organoids as personalized disease models [22, 53]. Consequently, patient-derived organoids could possibly not be accessible for all patients, but only be created for small groups of patients [63]. Thirdly, it could be difficult to translate the responses of organoids in the laboratory and reliably predict the efficacy of a drug in an individual patient. Although an organoid closely mimics with the original organ of the donor, it does not account for an entire body. Therefore, experts are skeptical about the type of evidence that an organoid generates and thereby about the added clinical value of this technology [57, 60, 63]. Several papers argued that clinical validation of organoid-based drug screening tests could be challenging especially in patients with rare diseases [57, 60, 63]. A potential solution given for this problem is to implement a ‘*n*-of-1’ trial: a single-patient randomized controlled trial with multiple crossovers to generate data about safety and efficacy of a drug for potential reimbursement [60]. Fourthly, the use of organoid technology for personalized drug screening and treatment selection implies a departure from the standard translational process, in which the safety of new drugs is tested in large cohorts of comparable patients. Consequently, potential risks might not be detected. Fifthly, it is currently not ensured that patients will gain access to novel therapies that are developed through the usage of ‘their’ organoids [68], since reimbursement policies differ between countries, and laboratory tests or treatments may not be offered to patients [67]. This is problematic, because, as said, the expectation of personal therapeutic benefits is seen by patients as one of the main reasons for participation in organoid research [55, 61, 66], but cannot (yet) be guaranteed. The new type of evidence resulting from personalized drug screening in patient-derived organoids requires new policies for drug reimbursement by insurance companies, which have traditionally depended on evidence of safety and efficacy resulting from large clinical trials [30, 60–63]. Sixthly, patient-derived organoids could potentially blur the lines between research and clinical practice, which are normally subject to different ethical–legal frameworks [30, 52, 55, 60, 62, 63, 66, 67]. On the one hand, personalized drug testing in organoids may be viewed as research because it could provide insights into drug and disease mechanisms. On the other hand, it could be considered care, when patient-derived organoids become part of a treatment strategy for the individual patient and sources of potential clinically relevant information for clinicians. As different sets of regulations and oversight mechanisms have traditionally applied to research and care [30, 52], it is not clear what standards apply to clinicians, researchers, and industries involved in organoid-based personalized medicine [62, 66, 67]. As the current regulatory framework is not adequate, authors are encouraging new guidelines, ethical oversight bodies, and standard operating procedures to facilitate the integration of both research and care and to address the challenges raised by regulatory differences between countries [60, 66, 67]. Lastly, some articles discuss the ethical implications of the use of organoids as personalized drug-testing tools for patient donors, including positive effects on patients’ health and well-being, by informing clinical management [55, 61, 68]. On the other hand, the use of organoids in personalized drug testing is associated with a potential for informational and personal harms, such as those resulting from feedback of unsolicited findings or privacy breaches [55, 67, 68].

#### Transplanting organoids in humans

The second area of potential future clinical application of organoid technology is organ replacement therapies, in which organoids are envisioned as a source of potential functional tissues for transplantation [23]. As an example, liver organoids could be used to restore liver function in patients diagnosed with metabolic liver disease [60, 70]. Preclinical studies in animals suggest that liver organoids may be suitable for transplantation, also in human recipients, and may, someday, become a less invasive and a more immediately available alternative to deceased donor organs [24]. Moreover, the combination of organoids with gene-editing technologies could potentially produce repaired, healthy organoids from patients with genetic defects, that can be used for transplantation [60, 70]. Four articles in our sample mention organoid transplantation as ethically challenging, but do not discuss it further [22, 51, 52, 57]. Five articles concisely discuss the ethical challenges regarding organoid transplantation [24, 30, 60, 63, 68] as described below.

### Clinical trials

When transplantable organoids move to the clinic, it will be necessary to test the safety and efficacy of the products by following a traditional translational cascade, from fundamental research, to non-human animals, to first-in-human (FIH) clinical trials, to larger randomized controlled trials, each with appropriate ethical oversight [63]. Two ethical difficulties of testing organoid transplantation in FIH clinical trials by following the traditional translational cascade are mentioned in the literature. Firstly, it is presently impossible to move transplantable organoids from fundamental research to FIH trials, because of the current standards for study design that require FIH trials to yield benefits to participants [24, 30, 57, 60, 63, 68]. In FIH trials, risks should be minimized and the potential individual benefits and social value should be maximized [60]. Current evidence for potential benefits comes from animal models only, which do not guarantee individual benefits for human patients [30]. Some articles in our sample argued that the traditional cascade of FIH to phase 1 to phase 2, 3, and 4 clinical trials, in which toxicity and/or efficacy of new drugs is tested, may not be suitable for testing transplantable organoids in humans [24, 30, 52, 60]. Schneemann et al. [24] proposed that FIH transplantation organoid trials should combine safety and efficacy outcomes in a trial design that allows participants a chance at benefit. Secondly, there are concerns that FIH trials for transplantable organoids might not be justifiable due to safety issues. Compared to traditional pharmaceutical drug trials, the transplantation of laboratory-grown organoids is a more invasive and complex procedure [30, 57]. In fact, the concept of transplanting regenerated complete organs in patients is entirely new. Given the uncertainties and lack of evidence, authors argued that FIH organoid transplantation trials would expose participants to unnecessary and unjustified risks [57, 60]. In short, Bredenoord et al. emphasized that FIH organoid transplantation can be justified if the following conditions are met: The risks for participants are minimized and the benefits are maximized (1), a translational cascade is followed which yields sufficient evidence to mitigate potential uncertainties and safety issues (2), and the FIH trial design allows participants the chance at medical benefit (3). To this end, a combined safety and efficacy trial could prevent promising interventions from failing due to high-risk outcomes in safety studies and may give earlier access to novel interventions [30].

### Psychological and societal concerns of organoid transplantation

Transplantation of organoids in humans is expected to raise psychological and societal concerns [30, 61, 68], for recipients as well as for society at large. Organoid transplantation could have what is called ‘soft impacts,’ because it could potentially influence the experience or the quality of life of organoid recipients and it may affect the recipient’s perception of their composite body [61, 68]. In organoid transplantation, however, the recipient and the donor would likely be the same individual, which is different and possibly less distressing compared to traditional transplantation, in which the organ comes from a living or deceased other [30]. Last, the ways in which patients and the public give meaning to maladies of organs will be reshaped [68]. For example, the public no longer see organ failure as a deadly, life-treating, incurable disease but start thinking: For every failing organ, a new personalized organ could be developed in the laboratory.

### Ethical concerns specific to sub-types of organoids

#### Brain organoids

Forty-nine articles discuss the ethical issues around the creation of brain organoids or cerebral organoids [21, 22, 27–29, 43–50, 52, 54, 55, 57–59, 62–65, 68, 71–92]. Both terms are used interchangeably. In this review, we use the term brain organoid. Traditionally, the difficulty of accessing and manipulating neural tissue is a major practical obstacle toward the understanding of neuropsychological, psychiatric, and neuro-developmental disorders [29, 44, 50, 76, 93]. Earlier in vitro cellular techniques for modeling the human brain were perceived as inadequate, as earlier models lacked the complex architectural features of the developing brain, and atypical neuronal function is difficult to detect or evaluate [22, 44, 76, 81, 93]. Brain organoids could potentially overcome these limitations and could open up possibilities for studying the human brain and its associated diseases and are therefore seen as promising in vitro 3D models [22, 45, 48, 49]. The field has already achieved major progresses; brain organoids have been successfully used to model and examine the pathogenesis of Zika virus-induced microcephaly, idiopathic autism, virus-induced microcephaly, and schizophrenia (20,23,84,86,89,28,46,47,50,55,66,74,77). Similarly, brain organoids have improved our scientific knowledge about brain tumors [22, 29, 44], neurodevelopment, and neurodegeneration [28, 44, 45, 48, 57]. In the future, it may even be possible to repair brain circuitry after damage following traumatic brain injury, stroke, or surgical resection [29, 44, 45, 50, 52, 54]. Furthermore, various brain organoid models are under development where brain organoids are connected to other organoids, called ‘assembloids,’ which can provide information about the communication between the brain and other organs, such as the gut–brain relationship [54, 57, 71, 76, 81, 94].

Despite these advantages associated with recent developments in brain organoid technology, there are ethical concerns related to the idea of creating brain models using human-sourced cells, notably in relation to their capacity to establish neural networks and start spontaneous electrical activity in vitro and possibly to spark or support ‘consciousness’ [21, 22, 27–29, 42, 44–49, 52, 54, 55, 57, 58, 63–65, 68, 71–83, 86–92]. Fifteen papers discuss findings of a study by Muotri and colleagues [95], in which electroencephalogram (EEG) patterns in brain organoids are described as comparable to those in brains of premature babies [29, 45, 47, 54, 57, 64, 71, 76–78, 81, 82, 89, 92]. Some authors wonder whether ‘mature’ brain organoids could eventually attain sentience, respond to light, and have the capacity to feel pain [22, 27, 42, 44, 45, 47, 49, 54, 55, 57, 63–65, 72, 81–83, 86, 87, 89, 91, 92], or perhaps be developed to a point of attaining higher cognitive abilities, such as learning or retrieving memories [42, 47, 54, 64, 79]. To date, researchers have already explored techniques which could make brain organoids capable of interacting with the outside environment, for example by connecting brain organoids to controllable robotic bodies [96] and muscle tissue [97], and creating brain organoids that can react to light [98]. Fusions of cerebral, thalamic, and retinal organoids might render such models capable of constructing the full visual pathway and visual perception in vitro [54].

Further, authors have written that cognitively advanced brain organoids might acquire some degree of moral status [44, 46, 47, 49, 54, 57, 63–65, 71, 78, 79, 81, 82, 84, 88, 90, 92] when they develop consciousness. Although different perspectives exist on how to conceptualize consciousness and on its role in the attribution of moral status [88, 90, 92], it is generally acknowledged that when an entity has interests, it has moral status [46, 47, 82]. In addition, capacities such as consciousness, suffering, self-awareness, or sentience are tightly linked with—and arguably necessary for—moral status [44, 46, 47, 49, 54, 55, 57, 63, 64, 71, 78, 79, 81, 82, 88, 90]. A self-consciousness being can have interests, for instance, to pursue pleasure and avoid pain [99]. Therefore, when brain organoids develop consciousness, researchers should not treat brain organoids as biological material, but as entities with interests [47, 82, 88, 89, 92]. This means that some forms of experimentation using brain organoids might become unethical. In contrast, some experts argue that brain organoids can never develop consciousness, because of the absence of interaction with a (human) social environment in the laboratory [47, 65, 71, 78, 79, 82, 92]. For this reason, they believe higher moral status should never be attributed to them [71]. An empirical study, in which 28 persons with neurological diseases or psychiatric disorders and laymen were interviewed about the development, use, and storage of cerebral organoids in the Netherlands [55], suggests that respondents’ perceptions of the moral value of brain organoids are closely connected to their imaginings of what cerebral organoids look like, how they are created, and what sort of functions they have [55].

When organoids are seen as beings with moral status, this raises questions for donor consent and donor control over their ‘clones’ [54, 55, 65]. Will tissue donors still be able to withdraw consent when ‘their’ organoids develop consciousness [54]? Further research is suggested to understand how consciousness in brain organoids can be detected and possibly be prevented [28, 42, 46, 47, 49, 52, 54, 55, 57, 71, 78–83, 85, 86, 89, 90]. Despite the current lack of scientific consensus on the definition of consciousness [82], multiple researcher groups in our sample are enthusiastic about the idea of developing a sensitive, objective, noninvasive, standardized ‘consciousness test’ for brain organoids to screen them for advanced cognitive capabilities they could plausibly develop [22, 28, 44, 47, 54, 55, 71, 74, 76, 78, 79, 82, 85, 86, 89]. For instance, the Perturbational Complexity Index (PCI) is often mentioned as a possible screening tool for detecting consciousness in brain organoids [45, 54, 71, 74, 76, 78, 80, 82, 85], and gene-editing tools are suggested to prevent the development of consciousness in brain organoids [47].

Nevertheless, if brain organoids could potentially develop consciousness which can be detected (or not), how should these entities be treated in order to prevent unethical forms of experimentation without haltering valuable scientific knowledge? Four different approaches for monitoring brain organoid research are mentioned in the literature, which could change obligations and responsibilities of researchers toward brain organoids. Firstly, some authors have discussed that stringent ethical oversight might be needed for research using brain organoids [29, 44, 45, 47, 49, 52, 54, 55, 63, 65, 71, 78, 79, 81, 85, 89–91], for instance by specialized research ethics review boards [49, 55, 57, 65]. Researchers might be required, for instance, to reduce the total number of brain organoids wherever possible [85, 89]. Secondly, several authors have called for special ethical guidelines or (international) regulatory frameworks for clinical research using brain organoids to ensure the welfare of brain organoids in the future [29, 45, 47–49, 54, 57, 63, 65, 71, 76, 78, 80, 82, 85, 86, 90–92, 100]. For example, Koplin and Savulescu et al. [47] recently developed an ethical framework that sets more stringent research restrictions for advanced brain organoids than for organoids that cannot plausibly possess consciousness. Thirdly, brain organoid laboratory researchers, policymakers, and bioethicists are called upon to work together from (the early) stages of research and development onwards. Emerging ethical questions could be identified sooner, prompting researchers to take new directions [49, 54, 57, 65, 90, 91]. Finally, deliberations could be organized about the kinds of legal rights brain organoids should be granted [44, 45, 47, 49, 54, 65, 71, 82, 87, 89, 91], and whether it should be allowed to manipulate or genetically alter [45, 47, 49, 71], or destroy brain organoids [45, 47, 49, 71], or use them for clinical transplantation in humans [54]. In sum, the literature contains suggestions about special research ethics review boards, frameworks, or legislation to prevent unethical forms of experimentation using brain organoids—without halting valuable scientific discovery. At the same time, scientists emphasize that it is not likely that brain organoids will ever develop humanlike consciousness.

#### Chimeras

Thirty articles in our sample deal with transplantation of human-derived organoids into animals, and the creation, thus, of chimeras, which are defined as organisms composed of cells from two or more species [21, 22, 42, 44–47, 49–51, 53, 54, 57–59, 62–65, 68, 69, 71, 73, 76, 79, 81, 88, 92]. Transplantation into animals is done primarily to increase vascularization of organoids [49, 53, 54, 57, 58], thus enabling them to grow larger than their maximum achievable size in vitro, and leading to more representative models of human development and disorders [49, 101]. Despite these advantages, the idea of creating chimeras by transplanting brain organoids in animals leads to moral concerns, notably in relation to ‘humanization’ of the brains [21, 42, 46, 49–51, 53–55, 57, 58, 63–65, 69, 71, 73, 76, 79, 81, 88, 92]. These concerns are accompanied by discussions of a recent study from Mansour et al. in which brain organoids were transplanted in rodents (102). The organoid transplants in this study show advanced neural differentiation, gliogenesis, integration of microglia, and growth of axons to multiple regions of the brain. Imaging even showed functional neural networks and blood vessels in the organoid transplants [101]. It is feared that integration of human cells into the central nervous systems of animals might lead to ‘humanlike consciousness’ or ‘self-awareness’ and result in a morally ambiguous status of the chimera [22, 42, 46, 49, 50, 53, 54, 57, 58, 64, 65, 76, 81]. Chimeras might be said to have interests or might need to be due respect more than other animals, based on their human heritage [46, 50, 57, 88, 92]. Some authors have discussed, for instance, whether laboratory mice with advanced cognitive capacities should be destroyed at the end of a study [44, 49] or given special treatment, for instance only use them for important purposes [44, 46, 49, 64, 69, 71]. Some argue that research using chimeras should comply with more stringent guidelines and ethics review to ensure animal welfare [21, 22, 46, 49, 50, 54, 55, 57, 65, 73, 81, 88, 92], and some argue that chimeras might need some form of stewardship [44, 64], or assistance with decision-making about research participation [64]. In addition, another ethical concern mentioned is that when animals acquire cognitive or psychological/mental abilities that only human process, human dignity will be violated [54, 92].

Further research should focus on which brain enhancements of animals are morally concerning to society, how these enhancements should be recognized when they occur, and how they can be avoided [22, 46, 49, 50, 54, 57, 58, 79, 81, 88]. It have been suggested, for instance, that transplantation should be limited to a maximum number of human stem cells [79, 88], that animal behavior and physical change should be closely monitored [50, 57, 58, 79], or that the mirror test should be performed (i.e., observing the animal’s response to its mirror image) [50]. Some authors, however, believe that under current laboratory conditions, development of humanlike consciousness in animals is not likely, and thus, that it does not pose any serious ethical challenges to the advancement of organoid research [57].

Another concern is related to donor consent: Donors may disapprove of the creation of chimeras using (brain) organoids based on their tissue and thus withhold or withdraw their consent [55, 62, 64, 65, 68]. In the future, it has been suggested that donors should be informed about these applications as part of the informed consent process [44, 68]. Moreover, there are unresolved questions on legal ownership of enhanced or conscious chimeras [50, 54].

Finally, transplantation of human gonadal organoids, such as testicular or ovarian organoids, into animals, poses moral concerns about the potential for cross-species reproduction of human and non-human creatures, which, according to experts, should be avoided [58, 63].

#### Gastruloids

Sixteen papers mentioned the ethical acceptability of the usage of gastruloids in research [27, 30, 42, 51, 58, 62, 63, 73, 79, 80, 82, 102–105]. Gastruloids constitute a sub-type of organoids, but they are distinct because they do not copy organs but rather early developmental processes [58, 63, 103, 104]. Gastruloids are somewhat similar to human embryos, because they contain elements of the primitive streak formation and cells from each of the three germ layers, and they recapitulate aspects of early embryogenesis in vitro [63, 103–105], though their morphology is somewhat different [104]. Research with gastruloids could provide insights into early human embryo development, species–species differences, and disorders associated with first-trimester pregnancy and miscarriage [42, 58, 63, 103, 104].

To date, ethical concerns have been brought to the fore regarding the moral status that could be attributed to gastruloids [42, 46, 58, 62, 63, 68, 73, 82, 103–105], and the extent to which gastruloids may be permitted to mature [58, 63, 68, 73, 102–105]. The discussion ties in with the long-standing debate about the morality of creating human embryos or ‘early human life’ in vitro. In this debate, it is argued that human embryos should not be used in research virtue of the kinds of beings (i.e., persons) they might become in the future [46, 104]. In gastruloids, however, the potential to develop into a human being can be switched off by knocking out genes necessary for further embryonic development. When these genes are switched off, gastruloids can never qualify as embryos and do not need to be protected to the extent human embryos are usually protected [103, 104]. However, currently there is no universally accepted biological, legal, or ethical definition of the human embryo. This means that there are different answers to the question whether gastruloids should be classified as embryos, which further complicates the discussion about when a moral status should be attributed to gastruloids [103, 104].

Furthermore, in discussions on the extent to which gastruloids may be permitted to mature, reference is often made to the internationally well-accepted 14-day rule [58, 63, 68, 73, 104, 105]. This rule is endorsed by the Warnock committee in 1982 and limits researchers to culture human embryos for 14 days of development in vitro. It takes 14 days from fertilization for the primitive streak to appear [63, 73, 104, 106]. The formation of the primitive streak in human embryos is morally significant, because it represents what is called ontological individuation [58, 63, 73, 104, 105]. Before this point, twinning could occur: Embryos could split into two or fuse together. Therefore, it is argued that at 14 days, the embryo becomes a morally significant individual and is ‘destined’ to become a unique future person [63, 104, 106, 107]. Based on the similarities between gastruloids and human embryos, the 14-day rule seems fitting for culturing gastruloids in vitro. However, researchers are calling for an extension of the 14-day rule for gastruloids, as this would give them the opportunity to obtain important scientific insights in (disorders of) fertility [58, 63, 73, 103, 104]. At the same time, compared to human embryos, gastruloids can mimic the process of gastrulation (an early developmental process in which an embryo transforms from an one-dimensional layer into a multidimensional structure) in a shorter period of time [108], leading them to reach individuation sooner, which could be an argument to claim that the permitted days for developing gastruloids in vitro should be fewer than those for human embryos [58, 63, 104]. Finally, authors have emphasized the necessity of ethics oversight bodies and specific regulations and legislation [58, 63, 68, 73, 102, 105], notably in relation to 14-day rule, and of monitoring advances concerning gastruloid research that might necessitate new and more detailed ethical analyses [58, 63, 73, 103–105]. As an example, Pereira Daoud et al. [104] suggested to use a set of morally relevant features instead of a time limit to determine the extent to which gastruloids can be developed further. In addition, more conceptual research on embryo terminology and developmental maturity is called for to assess the morally relevant similarities and dissimilarities between gastruloids, human embryos, and other embryo-like entities [63, 73, 102–105].

## Discussion

To our knowledge, this is the first systematic review of the scientific and scholarly literature on ethical issues of organoids. This review includes all ethical issues mentioned in the literature on organoid technology, both issues mentioned only in passing and issues that are discussed more extensively.

A large part of the ethical literature on organoids is focused on the challenges of providing an appropriate informed consent model for tissue donors in organoid biobank research. One of the reasons why this is challenging is because of opposing interests between biobanks, commercial parties, and tissue donors [30, 58–60, 62, 66–68]. All four published empirical studies of the perspectives of patients, donors, and/or professionals on tissue donation for organoid biobanking [62, 66, 67, 109] show that tissue donors wish to stay informed about how their tissue will be used, out of curiosity, a preference to have a degree of control or be able to withdraw, or a desire to know the results of research activities [55, 62, 66, 67, 109]. The two reasons reported in the literature about why donors want to retain control over the use of their tissue in organoid research are as follows: donors wish to know (or check) whether their contribution to science or medicine is meaningful (1), and donors experience a general distrust toward the involvement in research of commercial parties (2) [62, 66, 67, 109]. However, organoid biobanking for personalized medicine involves close cooperation with commercial parties. On the one hand, many professionals consider these partnerships crucial for making organoid biobank research financially viable [67]. On the other hand, many professionals also express uneasiness with companies generating profit using and commercializing tissue derived from patients with urgent health needs [67]. Distrust of commercial parties among donors is believed to come from the general concern that commercial parties will not use organoids in ways that are in the best interests of patients and society [62, 66, 67, 109]. The desire of donors to be in control, coupled with their concerns about commercialization, highlights that often-used models of broad consent are not fitting for long-term storage and open-ended future use of donor tissue in organoid (biobank) research [67]. Moreover, there are concerns that when broad consent is used for organoid research, donors might not be willing to donate their tissue anymore out of distrust, and that this may limit progress in organoid research [55, 67]. Therefore, a shared idea is to focus less on the protection of autonomy via (broad) informed consent and more on setting up a governance infrastructure and getting participants to understand the conditions under which their tissue and data are put to use in research [62, 66, 67, 109]. Therefore, in our opinion the ‘dynamic consent’ [110–112] or the ‘consent for governance’ [59, 113] would be appropriate models for tissue donors donating their tissue for organoid research (Table 2). These consent models aim to respect the preferences and values of patients as key stakeholders, rather than as passive donors, and are able to give them control by engaging them in an on-going communicative (governance) process [59, 113].

Another main finding is that special sub-types of organoids, notably brain organoids, are drawing by far the most attention in the ethical literature on organoids. This focus on brain organoids might be explained by the more extreme nature of this sub-type, which raises apparently more fascinating philosophical and ethical questions about consciousness than other, more ordinary organoids, such as gut organoids. A recent quantitative study by Ide et al. [114] showed evidence of an increase in societal interest in brain organoids in news reports over time. The number of news reports was three times higher during 2017–2020 compared to 2013–2016, which indicates an increasing public focus on the ethical aspects of brain organoid research [114], despite the fact that biomedical researchers and ethicists have stated that there are no indications that the structural complexity and functional ability of brain organoids will (ever) become such that consciousness and self-awareness becomes possible [45–47, 50, 57, 71, 75, 78, 79]. The overemphasis on brain organoids in the representation of organoid technology in popular media is concerning, because it is not clear how this influences public opinion, and it might lead to fears or concerns regarding organoids. Gilbert et al. [115] warn against inaccurate portrayal of new technologies in mass media, and the influence on the public this may have. When inaccurate ideas about applications of organoids are reinforced, this could potentially increase the ‘Yuck factor’ [114, 116]. Thus, prominent discussions on brain organoids may have influenced public perceptions and expectations of what organoids are and what organoids can be used for, which could potentially have negatively framed policy debates about organoids [25, 64, 75]. Researchers should be aware of these effects [115]. They may need to direct their efforts at addressing and correcting inaccurate public perceptions on organoids to promote meaningful public engagement. Further empirical research on public perspectives on (brain) organoids may help to identify knowledge gaps and misunderstandings.

At the same time, other sub-types of organoids, including gonadal organoids, and ethical debates about potential clinical applications have been somewhat neglected in the current body of literature. As gonadal organoid technology may increase the possibilities of male and female reproduction, as well as reproduction of other lifeforms, including human–animal chimeras [63], it merits ethical consideration. We encourage future research efforts to be directed at more in-depth investigation of ethical issues related to gonadal organoids. Also, the incomplete representation of potential future applications of organoid technologies is striking, as these technologies might soon be ready for clinical trials and implementation in the clinic, raising very real and practical ethical questions. Only five articles mention ethical issues surrounding the use of organoids for personalized medicine [57, 60, 61, 63, 68]. These issues are discussed only briefly in subsections of those five articles, unlike brain organoids, about which entire articles and reviews have been written.

There are even fewer empirical studies exploring ethical issues from the perspectives of stakeholders involved in research or clinical applications of organoids. This gap in the literature is especially pressing for organoid transplantation, for three reasons. Firstly, interview studies and focus groups that investigate the perspectives of patients and professionals regarding organoids as potential clinical application found evidence that transplantation is perceived as a ‘sensitive application’. For instance, two studies reported that most respondents wish to stay away from ‘unworthy,’ ‘trivial,’ or ‘sensitive’ applications, such as growing whole organs or transplantation [61, 66]. Although the reasons why, and to what extent, stakeholders are concerned about the future technical possibilities of organoid transplantation are not mentioned in these papers, it will be important to understand these concerns, so they can be addressed to facilitate the development and implementation of organoid technology in transplantation care in the future [52, 60, 117, 118]. Secondly, the perceptions of potential recipients on organoids for transplantation are currently unknown. When an organoid is used for transplantation, it will become an integral part of a recipient. Further studies should investigate whether prospective recipients will accept organoids for transplantation and what the implications of organoid transplantation will be for their well-being and self-perception. Lastly, all the empirical data available on patient perspectives come from studies conducted in the Netherlands by the same research group and mainly focused on one disease area (CF). Therefore, we encourage researchers from other countries to complement the Dutch findings and extend focus on other organoid sub-types and other clinical applications, notably organoid transplantation. As several European Union-funded projects, such as the ‘VANGUARD’ (https://vanguard-project.eu) or the ‘ORGANTRANS’ (https://organtrans.eu) project, are already underway to develop transplantable bioartificial organs or organoids, we expect that several FIH clinical trials will be initiated in the coming years. More detailed analysis of ethical issues surrounding organoid transplantation is urgently needed, so that guidance can be developed for research groups involved in the clinical development of organoids for transplantation.

The one discussion that did take place around organoid transplantation is mainly focused on ethical challenges surrounding first-in-human (FIH) trials [24, 30, 60, 63]. Concerning FIH clinical trials, one might argue that FIH trials have already started. For example, the FDA has recently granted Fast Track Designation for VX-880—the first investigational stem cell-derived therapy utilizing organoids for the treatment of type 1 diabetes through their infusion in the liver [119]. We identified five ethical aspects of FIH trials that warrant further discussion: (1) unanticipated events could occur because the concept of transplanting organoids is new; (2) the evidence to predict the risks and benefits of this application in humans is lacking; (3) choosing the most appropriate study population for organoid transplantation is challenging; (4) transplantation requires an invasive procedure, in contrast to traditional drug trials, therefore a different ethical approach is needed; and (5) choosing the right study design with the right choice of outcomes and comparators is a difficult ethical and legal task [24, 30, 60, 63]. We found several concrete recommendations to organize organoid transplantation clinical trials in an ethically responsible way [24, 63]: (1) a combined safety and efficacy trial instead of phase 1–4 clinical trials (i.e., participants are given an expected therapeutic dose, and efficacy is added as an end point); (2) an ethics committee that can decide whether the preclinical evidence is convincing enough to guarantee a claim of potential individual benefits for participants in FIH transplantation trials; (3) lifelong follow-up for participants; (4) a voice for participants on the risk–benefits balance, informed consent, and patient selection for clinical trials; (5) strict attention to informed consent and assent; and (6) a proactive interdisciplinary dialogue between scientists, policymakers, ethicists, the public, research participants, and clinicians to stimulate responsible clinical research and innovation in the field of organoid transplantation.

Finally, for the application of organoid technology in personalized medicine, we found that the following three ethical issues are important: (1) accessibility for all patients, (2) maintaining linkage between patients and their organoids when the latter are used for (research in the area of) personalized medicine, while safeguarding the privacy of organoid donors, and (3) adapting existing models for obtaining clinical evidence for marketing authorization and reimbursement, so that these models can accommodate the new types of evidence provided by organoids. Implementation of ‘*n*-of-1’ trials [60], new drug reimbursement policies, and suitable infrastructures to return valuable results to patients are suggested as conditions for responsible implementation of organoid technology in personalized medicine [30, 60–63].

### Limitations and strengths

First, the large degree of interdisciplinarity in the ethical literature, and concurrent variation in terminology, publication standards and journals, made it challenging to search all papers mentioning ethical issues related to organoids. However, for our systematic review we used (extensive) search strings adapted to the requirements of each database, and therefore the chance of missing relevant literature is limited. Therefore, we consider it unlikely that we have missed relevant scientific papers for inclusion. Second, it is challenging to systematically include the ethical literature, because explicit guidelines for systematic ethical reviews are lacking. The only study on this topic have been done by Mertz et al. [41] who have reported trends in the quality of published systematic and semi-systematic reviews of ethical literature, and suggested that the PRISMA statement is applicable to most elements of the selection of ethical literature. This is why we have also used this statement for our systematic review. The same study emphasized that elements of analysis and synthesis of literature are even less standardized. Therefore, we used the recommendations of Mertz et al. [41] to report the following results: the information unit, technical procedure to extract the relevant information, and the method for synthesis of ethical issues. Last, the analysis and synthesis were conducted by only one researcher (DJ). Since the analysis may be influenced by the background and thus subjectivity of the researcher, two other researchers (EB and EM) first did a critical review of the analysis procedure to reduce research bias in the results. However, this does not guarantee that there is no research bias at all. Additionally, to support evidence-based health care meta-research, conceptual analysis and interdisciplinary discussion are needed to develop a valid standardized guideline especially made for systematic reviews of ethical literature.

## Conclusions

In conclusion, organoid research is evolving rapidly. It will therefore be crucial to continue monitoring developments related to organoid technology that might necessitate new and more detailed ethical analysis. The purpose of monitoring would be to address potential opportunities and risks, without leading to unnecessary restrictions on organoid research. At this moment, the ethical literature on organoids seems disproportionately focused on special sub-types of organoids—notably, brain organoids, human–animal chimeras, and gastruloids. This leads to an inaccurate and incomplete representation of potential future organoids applications, which might have influenced public perceptions and ethical debate around organoids. More ethical research is needed in areas in which clinical applications are nearer to fruition. Very limited ethical discussion has been taken place, for instance, on the use of organoids in human transplantation and the implications of this application of organoid technology for patients, for the organization of health care and for society at large. Also, empirical research on donor and public perspectives in various settings is desired to improve understanding, stimulate responsible innovation, and address public concerns around organoids.

## Supplementary Information

**Additional file 1**: PRISMA statement.

**Additional file 2**: Searching strategy.

## Data Availability

All the data are presented in this manuscript.
