# Supplementary material for: Organoids: a systematic review of ethical issues
Source: Stem Cell Res Ther. 2022 Jul 23;13:337. doi: 10.1186/s13287-022-02950-9 (PMC9308907; doi:10.1186/s13287-022-02950-9)
Supplement: Supplementary file 2 — Additional file 2: Searching strategy. [file 13287_2022_2950_MOESM2_ESM.docx]

**Additional file 2 - Searching strategy**

**Embase**

('organoid'/exp OR 'organoid culture'/de OR (organoid* OR ((mini) NEAR/3 (organ OR organs OR brain*)) OR colonoid* OR enteroid* OR (organ* NEAR/1 dish*) OR gastruloid*):ab,ti,kw) AND ('ethics'/exp OR 'bioethics and professional ethics'/de OR 'moral status'/de OR 'patient right'/exp OR 'morality'/de OR 'social aspect'/de OR 'ethicist'/de OR (ethic* OR unethic* OR bioethic* OR bio$ethic* OR ((moral OR morale OR morals OR morales) NEAR/3 (status* OR stand* OR polic* OR medic* OR concern* OR obligation* OR duty OR duties)) OR ((patient OR patients) NEXT/1 (right OR rights OR autonom*)) OR confidentialit* OR informed-consent* OR misconception* OR morality OR immoral* OR equipois* OR ((social) NEAR/3 (aspect* OR factor*))):ab,ti,kw)

**Medline**

(Organoids/ OR (organoid* OR ((mini) ADJ3 (organ OR organs OR brain*)) OR colonoid* OR enteroid* OR (organ* ADJ1 dish*) OR gastruloid*).ab,ti,kf.) AND (exp Ethics/ OR exp Patient Rights/ OR Morals/ OR ethics.fs. OR (ethic* OR unethic* OR bioethic* OR bio-ethic* OR ((moral OR morale OR morals OR morales) ADJ3 (status* OR stand* OR polic* OR medic* OR concern* OR obligation* OR duty OR duties)) OR ((patient OR patients) ADJ (right OR rights OR autonom*)) OR confidentialit* OR informed-consent* OR misconception* OR morality OR immoral* OR equipois* OR ((social) ADJ3 (aspect* OR factor*))).ab,ti,kf.)

**Cochrane**

((organoid* OR ((mini) NEAR/3 (organ OR organs OR brain*)) OR colonoid* OR enteroid* OR (organ* NEAR/1 dish*) OR gastruloid*):ab,ti,kw) AND ((ethic* OR unethic* OR bioethic* OR bio$ethic* OR ((moral OR morale OR morals OR morales) NEAR/3 (status* OR stand* OR polic* OR medic* OR concern* OR obligation* OR duty OR duties)) OR ((patient OR patients) NEXT/1 (right OR rights OR autonom*)) OR confidentialit* OR informed-consent* OR misconception* OR morality OR immoral* OR equipois* OR ((social) NEAR/3 (aspect* OR factor*))):ab,ti,kw)

**Web of Science**

TS=(((organoid* OR ((mini) NEAR/2 (organ OR organs OR brain*)) OR colonoid* OR enteroid* OR (organ* NEAR/1 dish*) OR gastruloid*)) AND ((ethic* OR unethic* OR bioethic* OR bio$ethic* OR ((moral OR morale OR morals OR morales) NEAR/2 (status* OR stand* OR polic* OR medic* OR concern* OR obligation* OR duty OR duties)) OR ((patient OR patients) NEAR/1 (right OR rights OR autonom*)) OR confidentialit* OR informed-consent* OR misconception* OR morality OR immoral* OR equipois* OR ((social) NEAR/2 (aspect* OR factor*)))))

**PsycInfo**

((organoid* OR ((mini) ADJ3 (organ OR organs OR brain*)) OR colonoid* OR enteroid* OR (organ* ADJ1 dish*) OR gastruloid*).ab,ti.) AND (exp Ethics/ OR exp Client Rights/ OR exp Morals/ OR (ethic* OR unethic* OR bioethic* OR bio-ethic* OR ((moral OR morale OR morals OR morales) ADJ3 (status* OR stand* OR polic* OR medic* OR concern* OR obligation* OR duty OR duties)) OR ((patient OR patients) ADJ (right OR rights OR autonom*)) OR confidentialit* OR informed-consent* OR misconception* OR morality OR immoral* OR equipois* OR ((social) ADJ3 (aspect* OR factor*))).ab,ti.)

**Google Scholar**

organoid|organoids|colonoid|enteroid|gastruloid ethics|ethical|unethical|bioethics|moral|morale|morals|morality|immoral
